# A randomised trial of a medium-chain TAG diet as treatment for dogs with idiopathic epilepsy

**DOI:** 10.1017/S000711451500313X

**Published:** 2015-09-04

**Authors:** Tsz Hong Law, Emma S. S. Davies, Yuanlong Pan, Brian Zanghi, Elizabeth Want, Holger A. Volk

**Affiliations:** 1 Department of Clinical Science and Services, Royal Veterinary College, Hatfield AL9 7TA, UK; 2 Section of Computational and Systems Medicine, Imperial College, London SW7 2AZ, UK; 3 Nestlé Purina Research, St Louis, MO 63164, USA

**Keywords:** Epilepsy, Ketogenic diets, Medium-chain TAG, Seizures

## Abstract

Despite appropriate antiepileptic drug treatment, approximately one-third of humans and dogs with epilepsy continue experiencing seizures, emphasising the importance for new treatment strategies to improve the quality of life of people or dogs with epilepsy. A 6-month prospective, randomised, double-blinded, placebo-controlled cross-over dietary trial was designed to compare a ketogenic medium-chain TAG diet (MCTD) with a standardised placebo diet in chronically antiepileptic drug-treated dogs with idiopathic epilepsy. Dogs were fed either MCTD or placebo diet for 3 months followed by a subsequent respective switch of diet for a further 3 months. Seizure frequency, clinical and laboratory data were collected and evaluated for twenty-one dogs completing the study. Seizure frequency was significantly lower when dogs were fed the MCTD (2·31/month, 0–9·89/month) in comparison with the placebo diet (2·67/month, 0·33–22·92/month, *P*=0·020); three dogs achieved seizure freedom, seven additional dogs had ≥50 % reduction in seizure frequency, five had an overall <50 % reduction in seizures (38·87 %, 35·68–43·27 %) and six showed no response. Seizure day frequency were also significantly lower when dogs were fed the MCTD (1·63/month, 0–7·58/month) in comparison with the placebo diet (1·69/month, 0·33–13·82/month, *P*=0·022). Consumption of the MCTD also resulted in significant elevation of blood *β*-hydroxybutyrate concentrations in comparison with placebo diet (0·041 (sd 0·004) *v.* 0·031 (sd 0·016) mmol/l, *P*=0·028). There were no significant changes in serum concentrations of glucose (*P*=0·903), phenobarbital (*P*=0·422), potassium bromide (*P*=0·404) and weight (*P*=0·300) between diet groups. In conclusion, the data show antiepileptic properties associated with ketogenic diets and provide evidence for the efficacy of the MCTD used in this study as a therapeutic option for epilepsy treatment.

Epilepsy is a common chronic neurological disorder in humans and dogs, with an estimated prevalence in dogs of 1–2 %^(1)^ in a referral hospital population and 0·6 %^(2)^ in first-opinion practice. Higher prevalences up to 18 %^(^ ^3^ ^)^ have been reported in breed-specific studies with up to 33 % seen in certain families^(^ ^4^ ^)^. Epilepsy is characterised by recurrent epileptic seizures caused by abnormal, excessive, synchronous neuronal firing patterns^(^ ^5^ ^)^. Epilepsy has been associated with increased risk of premature and unexpected death, injuries, cognitive deterioration, neurobehavioural dysfunction and reduced quality of life (QoL)^(^ ^6^ ^–^ ^8^ ^)^. Despite ongoing research in understanding the pathophysiological manifestation of seizures and epilepsy, the cellular mechanisms remain elusive. As a result, approaches towards antiepileptic therapy are usually directed towards the control of seizures, most commonly chronic administration of antiepileptic drugs (AED), rather than prevention of epileptogenesis or comorbidities. Despite appropriate AED treatment, approximately one-third of dogs and humans with idiopathic epilepsy continue to experience seizures that are difficult to control^(^ ^9^ ^–^ ^11^ ^)^. Furthermore, AED-related side-effects such as ataxia, polyphagia, polyuria, polydipsia and incontinence in dogs as well as behavioural, sedative, cognitive or psychiatric adverse reactions in humans also contribute to reduction in QoL^(^ ^12^ ^,^ ^13^ ^)^. This emphasises the importance of new treatment strategies to improve the welfare of people with epilepsy.

A myriad of anecdotal reports and some published literature have suggested the importance of dietary manipulation in seizure management^(^ ^14^ ^)^. In particular, the ketogenic diet (KD) has been proposed as an alternative treatment strategy for canine epilepsy^(^ ^15^ ^)^. The ‘classic’ KD consisting of high fat, low protein and low carbohydrate, typically with ratios of up to 4:1 fats to proteins and carbohydrates, was first introduced in the 1920s for use in patients with childhood epilepsy^(^ ^16^ ^)^. Wilder initially suggested the use of the KD in order to mimic the metabolic state and biochemical changes associated with fasting, as fasting was shown to possess anticonvulsant properties^(^ ^17^ ^)^. A randomised controlled trial on childhood epilepsy showed promising results with 38 and 7 % of children on KD diets having >50 and 90 % seizure reduction, respectively. In comparison, only 6 % of the children on control diets achieved >50 % seizure reduction, with no children achieving >90 % seizure reduction^(^ ^18^ ^)^. Due to the antiepileptic effectiveness observed in the ‘classic’ KD^(^ ^19^ ^–^ ^22^ ^)^, other KD, some with improved palatability and diet toleration, have been proposed. These include the modified Atkins diet^(^ ^23^ ^)^, medium-chain TAG ketogenic diet (MCTKD)^(^ ^24^ ^,^ ^25^ ^)^, low-glycaemic-index treatment^(^ ^26^ ^)^ and diets that involve intermittent energitic restriction^(^ ^27^ ^)^. The conventional MCTKD is based on high proportions, 60 % or greater daily calories, of dietary fats in which at least 30 % and up to 60 % consists of medium-chain TAG (MCT)^(^ ^19^ ^,^ ^24^ ^,^ ^25^ ^)^. Such MCTKD diets, containing high proportions of dietary fats, have still been perceived as problematic due to palatability and diet restrictiveness.

The MCTKD utilise medium-chain (C6–C12) fatty acids, with the main constituents being octanoic and decanoic TAG, as an alternative fat source rather than long-chain TAG (LCT) implemented in the ‘classic’ KD^(^ ^26^ ^)^. In comparison, MCT in humans and dogs are more efficiently digested and absorbed via the gastrointestinal tract, and the resulting medium-chain fatty acids are transported by the portal vein to the liver where they are subsequently metabolised and converted primarily into ketone bodies^(^ ^28^ ^,^ ^29^ ^)^. Consequently, MCT are thought to be more ketogenic, producing higher ketone yields per kilocalorie of dietary energy in comparison with LCT^(^ ^30^ ^)^. However, a randomised trial comparing a MCTKD and the ‘classic’ KD, over a period of 3, 6 and 12 months in 145 children with intractable epilepsy, showed no significant differences in responder rates, seizure frequency reduction and tolerability between the diets^(^ ^25^ ^)^. Other studies evaluating the effectiveness of MCTKD have also shown anticonvulsant properties to varying degrees^(^ ^31^ ^,^ ^32^ ^)^.

Despite the unknown mechanisms of action of the KD, the antiepileptic properties of the KD in children have led to its proposal as a new treatment option for canine epilepsy. Recently, a novel diet with relatively low MCT levels (medium-chain TAG diet (MCTD)) developed for canine consumption was shown to be ketogenic and useful as a dietary strategy for cognitive enhancement in aged dogs^(^ ^33^ ^)^. This MCTD inherently is a potential candidate to investigate its effects on epilepsy. It is thought that canine epilepsy is not only naturally occurring but also similar in aetiology, heterogeneity, clinical manifestations and pathology to its human counterpart, and thus acts as a good translational model^(^ ^34^ ^,^ ^35^ ^)^. The primary aim of this study was to determine the antiseizure efficacy of the ketogenic MCTD in dogs with idiopathic epilepsy compared with a standardised placebo control diet.

## Methods

This study was conducted in accordance with the guidelines laid down in the International Cooperation of Harmonization of Technical Requirements for Registration of Veterinary Products (VICH) GL9 Good Clinical Practices (GCP) and the European Agency for the evaluation of Medical Products (EMEA). The study protocol was approved by the local Ethics and Welfare Group (EWG) (URN 2011 1132). The diet allocations were randomised and only available to the study nurse who was also the diet dispenser; therefore, dog owners, investigators and statisticians involved were blinded throughout the study. Furthermore, owners were also blinded from the content of both the MCTD and the placebo diet and were unaware that the study involved KD.

### Study design

The present study comprised of a 6-month prospective, randomised, double-blinded, placebo-controlled, cross-over dietary trial comparing the MCTD to a standardised placebo diet for canine epilepsy. Dogs were fed either the MCTD or the placebo diet for 3 months (day 1 to day 90±2 d) followed directly by a subsequent respective switch of diet for a further 3 months (day 90 to day 180±2 d). Relevant data were collected on the following variables at visit 1 (day 2), visit 2 (day 90 (sd 2) d) and visit 3 (day 180 (sd 2) d): seizure frequency (generalised seizures); body weight; measurements of serum phenobarbital (PB) and/or potassium bromide (KBr) concentrations as appropriate; dynamic bile acids; complete blood cell count; standard clinical serum chemistry; adverse events; and visual analogue score (VAS) for ataxia, sedation and QoL.

### Participants

Participants were recruited through different media in the UK based on the following inclusion criteria that were verified by telephone interviews with owners, followed by physical and laboratory examinations: canine species of mixed or purebred breeds; suspected to have idiopathic epilepsy (unremarkable former MRI scan); aged between 6 months and ≤12 years; weighing between 4 and ≤65 kg; no clinically significant findings on haematology and biochemistry or dynamic bile acid results; have unremarkable interictal neurological examinations for a dog on antiepileptic treatment; only one dog per household enroled to the study; have had at least three seizure episodes in the previous 3 months before the start of this study; on at least one antiepileptic treatment; and no use of drugs that could influence the metabolism of PB and KBr. Dogs intended for breeding in <2 weeks from the start of study; with known cause of epilepsy such as brain neoplasm, brain trauma, encephalitis and meningitis; with chronic or acute renal, hepatic or cardiac failure; with an acute or surgical condition at the time of enrolment; and bitches known or suspected to be pregnant or lactating were all excluded from the study.

Owners of dogs meeting all the above-mentioned criteria received further information, both verbally and in writing, on all aspects of the study, including information about the protocol and explanations about the seizure frequency diary that was to be completed at home. Dogs were enroled as an individual experimental unit and independent of other dogs. A unique study case number, consisting of a two-digit number, ascending in a chronological order of enrolment, was allocated and used to identify each dog on all documents and samples throughout the study. Twenty-one dogs were included in the analysis and all of them completed the study.

### Visual analogue score

VAS was recorded for ataxia, sedation and QoL using a line ranging from 0 to 100 mm. The observer was asked to draw a secondary intersecting line perpendicular to the line of measurement that best represented the subjective severity. A perpendicular line at 0 mm represented ‘asymptomatic/normal’ and at 100 mm represented either ‘ataxia so severe dog is unable to walk’ or ‘sedation to the extent dog only sleeps’ or ‘QoL is so poor euthanasia is requested’, respectively.

### Experimental food

The experimental placebo and test formulae were dry extruded kibble (Nestle Purina PetCare) formulated to meet or exceed the nutritional guidelines established by the Association of American Feed Control Officials (AAFCO). Both formulae were of the same ingredient composition, and formulated to contain <10 % moisture, at least 28 % crude protein, at least 15 % crude fat and 50 % carbohydrates with <2 % as crude fibre. The only compositional exception was that zero MCT were added to the placebo formula, and lard was used as fat substitute to ensure that the formulae were isoenergetic (1560·6 kJ/100g (373 kcal/100 g)), whereas the test formula contained 5·5 % MCT. MCT content was about 10 % of the total formula calories (based on fat as 35·6 kJ/g (8·5 kcal/g) and MCT as 28·5 kJ/g (6·8 kcal/g)). Proximate analysis of both formulae indicated that they were of similar composition, with the exception of MCT, as the placebo diet was void of C12, C10 and C8 fatty acids (each <0·100 % of placebo formula). Both formulae exceeded the AAFCO minimum requirements for essential fatty acids. The dogs were housed and fed mainly once or twice daily at home with no restrictions on water consumption. The owners were educated to keep the diet consistent throughout the study period. The amount of food given per day was calculated according to the weight of each dog in order to provide sufficient food to fulfil their nutritional requirements. A deviation of ±10 % food consumption (kg) was allowed to account for the individual needs of each dog, taking into consideration differences in activity level and physical condition. Dogs were restricted to consumption of the study diet, and thus treats or snacks were replaced by the respective placebo or MCTD food.

### Ketone body measurements

Three sets of blood samples were collected from the dogs over the duration of the study on the days of visit (baseline – day 2, end of period 1 – day 90 (sd 2) d and end of period 2 – day 180 (sd 2) d). Blood samples were collected 2 h after consumption of the respective diets and routine concomitant AEDs. Blood samples were collected and stored using clotting activator dipotassium EDTA-containing (with serum-separation gel) polypropylene blood-collection tubes and plain polypropylene blood-collection tubes (International Scientific Supplies Ltd). Blood samples were allowed to clot and serum was stored at −80°C. Serum samples were analysed for *β*-hydroxybutyrate (BHB) concentrations using an enzymatic end point (colorimetric) reaction assay as directed by following the kit manufacturer’s protocol (C444-0A; Catachem Inc.) and using an Olympus AU 640e (Beckman Coulter) chemistry analyser for measurements.

### Adverse events and concomitant treatments

Any abnormal health observation that was unfavourable, unintended and occurred after enrolment, regardless of whether it was considered as an MCTD- or a placebo diet-related event, was reported to the investigator and documented (online Supplementary Table S1). Details of the adverse event include the following: a description of the adverse event; length of the adverse event recorded in days; severity of the adverse event recorded using 1=mild, 2=moderate, 3=severe and 4=serious; frequency of the adverse event measured using 1=once, 2=occasionally, 3=regularly and 4=ongoing; concomitant treatment noted as 1=none and 2=yes; and final outcomes consisted of 1=resolved without further effects, 2=resolved with further effects, 3=unchanged, 4=euthanasia and 5=death. In light of the objectives of this study, seizure occurrences during the study period were not regarded as an adverse event. Any concomitant treatments including routine AED administered during the study period were recorded, detailing indication of treatments, products used and length of treatments (online Supplementary Table S2). To avoid confounding influences, concomitant AED medication and dosages were unchanged throughout the study. Eighteen of the twenty-one dogs were drug-resistant. All twenty-one dogs received PB. Most dogs were treated additionally with KBr (*n* 18). Some dogs were chronically treated with a third AED, imepitoin (*n* 1) or levetiracetam (*n* 4). Twelve owners had rectal diazepam or levetiracetam for pulse therapy at home available for the acute treatment of cluster seizure episodes.

### Statistical analysis

Seizure frequency refers to the number of seizures per month, and seizure day frequency refers to the number of days in a month with seizure occurrence. The severity of seizures was analysed using the McNemar test by comparing the presence of cluster seizures between diet groups. Comparisons between the MCTD and placebo-standardised diet groups were made using match-paired Student’s *t* tests for parametric data and Wilcoxon matched-pairs signed rank test for non-parametric data. The relationships between two variables, such as seizure frequency and age, were analysed using Pearson’s correlation coefficient analysis. All comparisons were two-sided, and *P*<0·05 was considered significant. Non-parametric data are presented as median (25th–75th percentile), and parametric data are represented as mean values and standard deviations.

Patterson *et al.* ^(^ ^36^ ^)^ performed power calculations using the results that were acquired from their study, which showed that twenty-two dogs in each group would be sufficient to show significant differences between diet groups using seizure frequency as the major outcome variable. We report only twenty-one dogs as one dog was excluded due to an error in the diet dispensed, which came to our notice only during data analysis after completion of the study.

## Results

### Study population

This study included twenty-one dogs of seventeen different breeds including the following: American bulldog, two Beagles, two Border Collies, Boxer, Cavalier King Charles Spaniel, English Bull Terrier, English Springer Spaniel, German Shepherd, Golden Retriever, Lhasa Apso, Mastiff, Rhodesian Ridgeback, Saint Bernard, Siberian Husky, Slovakian Rough Haired Pointer, Welsh Springer Spaniel, and three cross breeds (online Supplementary Table S3). The study population consisted of fifteen males, of which ten were neutered and five were intact, and six females, of which four were neutered and two were intact (online Supplementary Table S3). The dogs had a mean of 4·59 (sd 1·73) years of age and weighed a mean of 29·79 (sd 14·73) kg at the start of the trial (online Supplementary Table S3). There were no differences for the acute or chronic treatment regimens between the placebo and MCTD phases.

### Effects on seizure frequency, seizure day frequency and severity of seizures

**Fig. 1:**
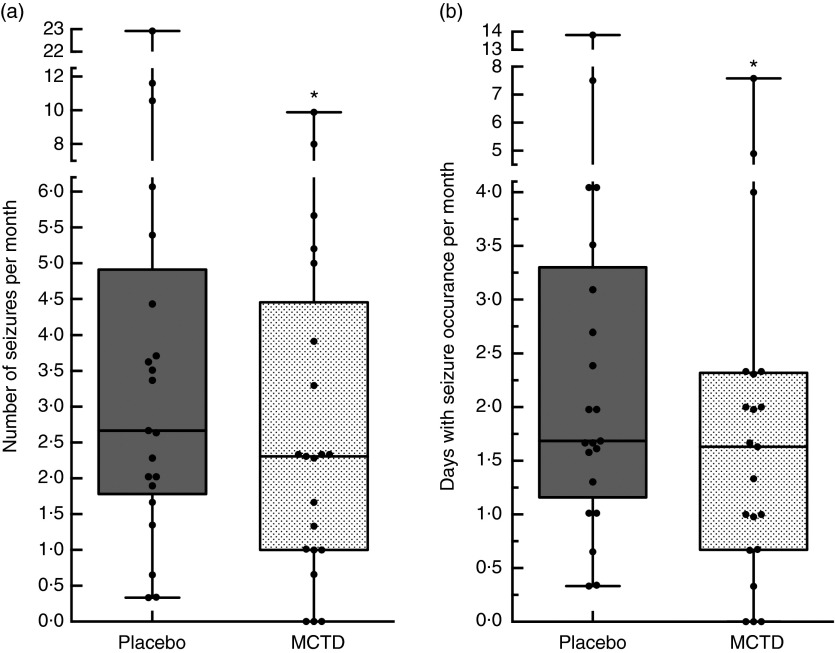
Effect of the medium-chain TAG diet (MCTD) on (a) seizure frequencies per month and (b) seizure days per month compared with the placebo-standardised diet (*n* 21). There were significant reductions in the (a) number of seizures per month (*P*=0·0195) and (b) seizure days per month (*P*=0·0216) during the MCTD phase in comparison with the placebo diet. Data are shown as box-and-whisker plots (central lines of the box represent the median, lower and upper limits of the box represent the 25th and 75th percentiles and whiskers represent the minimum and maximum). Two-sided Wilcoxon’s matched-pairs rank tests were used to compare placebo and MCTD groups. * *P*<0·05.

The results revealed a significantly lower seizure frequency when dogs were fed the MCTD (2·31/month, 1–4·46/month) in comparison with the placebo diet (2·67/month, 0·33–4·91/month, *P*=0·020; Fig. 1(a)). Three dogs achieved complete seizure freedom (100 % reduction), seven additional dogs had a 50 % or greater reduction in seizure frequency (56·85 %, 50·76–62·8 %) and five dogs had an overall reduction in seizure frequency (38·87 %, 35·68–43·27 %) (online Supplementary Table S4). Six dogs showed no response to the MCTD with an overall increase in seizure frequency. Seizure day frequency was also shown to be significantly lower when dogs were on the MCTD in comparison with the placebo diet (*P*=0·022), with over 80 % of the trial population achieving reduction in seizure day frequency (Fig. 1(b)) (online Supplementary Table S4). During the MCTD trial phase, three dogs achieved complete seizure freedom (100 % reduction), four dogs had over 50 % reduction in seizure day frequency (67·15 %, 58·25–75·15 %) and ten dogs had <50 % reduction in seizure day frequency (34·10 %, 19·40–43·45 %). Four dogs showed no response to the MCTD with an overall increase in number of seizure day frequency.

**Fig. 2:**
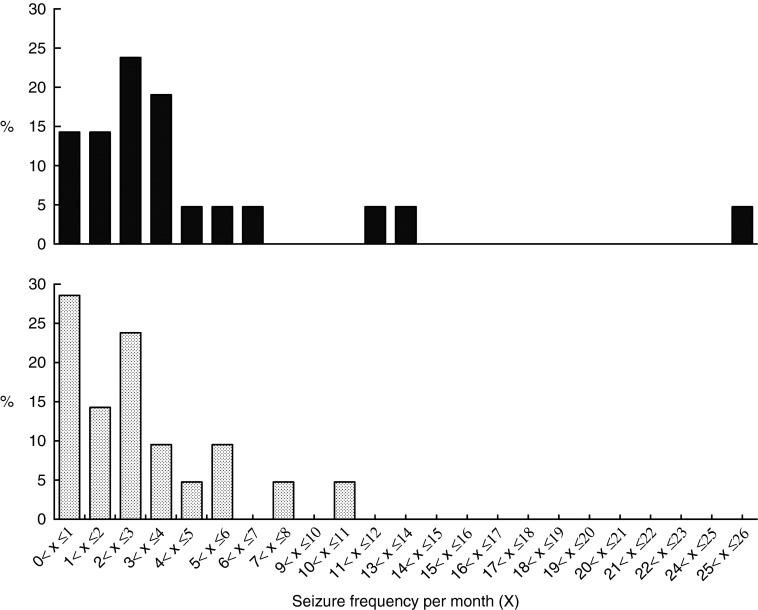
Effect of the medium-chain TAG diet (MCTD) on seizure frequency distributions compared with the placebo-standardised diet. The figure shows the distribution of the population (*n* 21) based on seizure frequencies per month, shown in a column bar graph. The MCTD resulted in higher percentages of the population experiencing lower seizure frequencies per month. 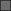, Placebo; 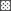, MCTD.

**Fig. 3:**
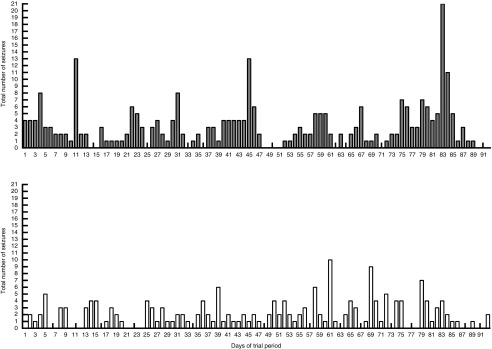
Effect of the medium-chain TAG diet (MCTD) on total number of seizures that occurred on each day throughout the study population (*n* 21) compared with the placebo-standardised diet. Total number of seizures was recorded over a period of 90 (sd 2) d for both the MCTD and the placebo diet. The figure shows a decrease in the number of seizures, seen on each day of the diet period, when dogs were on the MCTD in comparison with the placebo diet. 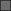, Placebo; 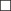, MCTD.

**Fig. 4:**
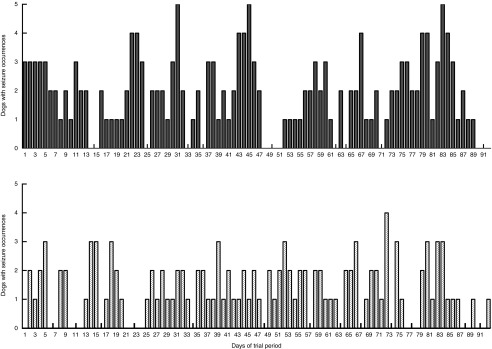
Effects of the medium-chain TAG diet (MCTD) on the number of dogs with seizure occurrence compared with the placebo-standardised diet per day of trial period. Occurrence of seizures per dog per day was recorded throughout the diet periods for both the MCTD and the placebo diet over a period of 90 (sd 2) d. The figure shows a reduction in the number of dogs in the population (*n* 21) with seizure occurrences in comparison with the placebo diet. 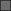, Placebo; 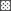, MCTD.

The MCTD resulted in a shift in the distribution of seizure frequencies per month with higher percentages of the whole population (*n* 21) experiencing reduced seizure frequencies compared with the placebo-standardised diet (Fig. 2). The total number of seizures that occurred in the study population (*n* 21) on each day of the MCTD period was also reduced in comparison with the placebo diet (Fig. 3). These results also show a reduction in the number of dogs with seizure occurrences during the MCTD period, with a stable distribution of around three dogs with seizure occurrences per day (Fig. 4). On the other hand, during the standardised placebo diet phase, the distribution was more varied with higher number of dogs with seizure occurrences per day. It is interesting to note that the results suggest a rapid onset of antiseizure efficacy. The total number of seizures (Fig. 3) and the number of dogs with seizure occurrences (Fig. 4) did not seem to gradually reduce throughout the MCTD phase, but instead showed improvements as early as day 1 and persisted throughout the diet period. There were no significant differences in the number of days with cluster seizures between diet groups (*P*=0·6171). There were also no significant associations between seizure frequency and seizure day frequency reductions with age, weight or BHB concentrations (online Supplementary Table S5).

### Effects on body weight, visual analogue score, serum antiepileptic drug concentration, complete blood count, clinical chemistry and blood ketone levels

**Fig. 5:**
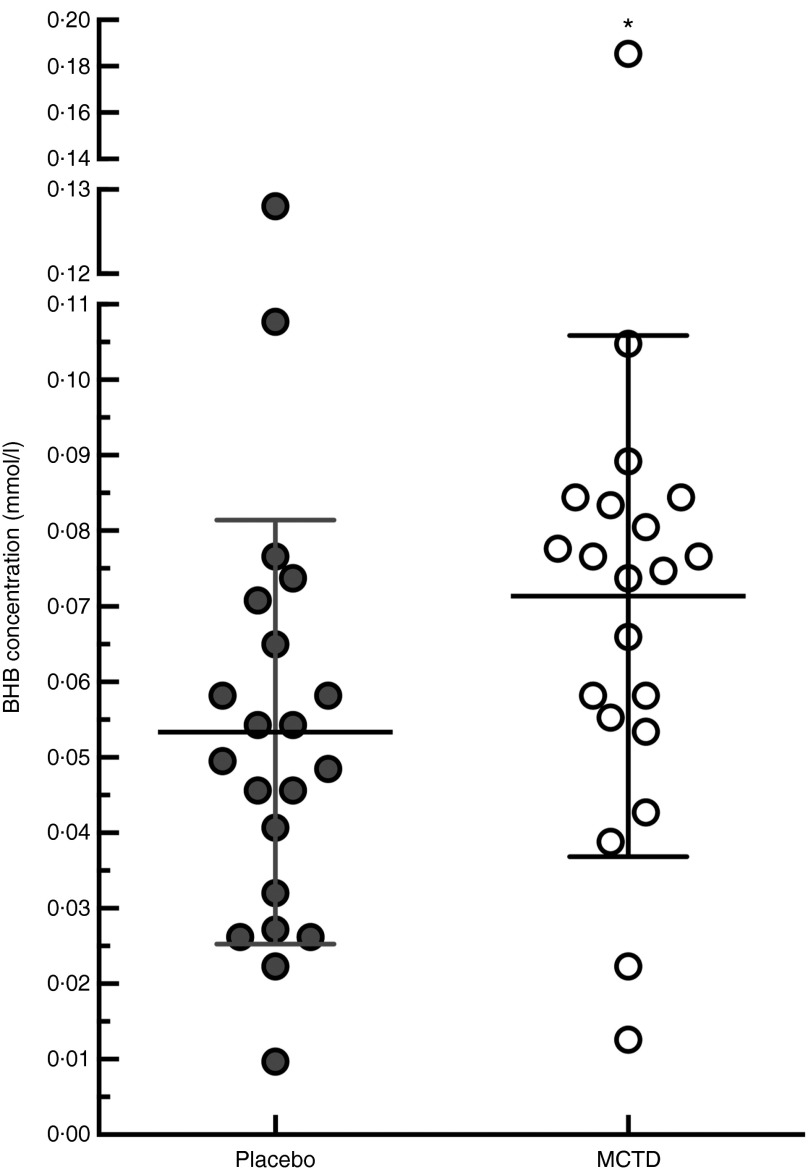
Effects of the medium-chain TAG diet (MCTD) on concentrations of *β*-hydroxybutyrate (BHB). BHB concentrations were measured after dogs (*n* 21) were fed the MCTD for a period 90 (sd 2) d and the placebo diet for a period 90 (sd 2) d. The figure shows a respective increase in BHB levels when dogs were on the MCTD in comparison with the placebo diet (*P*=0·0280). Data are shown as scatter plot (central line represents the mean values with standard deviations). Two-sided matched paired Student’s *t* test was used to compare the placebo and MCTD groups (* *P*<0·05).

There were no significant changes in serum concentrations of PB (26·50, 23·50–34·00 *v.* 32·50, 25·00–36·75 μg/ml, *P*=0·423) and KBr (1·23, 1·09–1·89 *v.* 1·29, 1·02–1·61 mg/ml, *P*=0·404) or weight (29·79 (sd 15·16) kg *v.* 29·61 (sd 15·51) kg, *P*=0·300) between the placebo-standardised diet and MCTD, respectively. There was a significant increase in blood concentrations of BHB when dogs were fed the MCTD (0·041 (sd 0·004) mmol/l) in comparison with the placebo diet (0·031 (sd 0·016) mmol/l, *P*=0·028) (Fig. 5). There were significant differences between placebo and MCTD groups in creatinine (83·62 (sd 17·08) μmol/l *v.* 79·67 (sd 15·02) μmol/l, *P*=0·025) and mean cell Hb concentrations (34·8, 33·5–35·0 *v.* 34·0, 33·0–34·7 g/dl, *P*=0·030), both of which were higher in the placebo diet group. All other complete blood count and clinical chemistry results, including glucose, were not significantly different between diet groups. There were no significant differences between diet groups in the VAS scores for ataxia (*P*=0·742), sedation (*P*=0·917) and QoL (*P*=0·568) (online Supplementary Fig. S1).

### Withdrawn dogs

All the aforementioned dogs did complete the study protocol. Ten further dogs had been recruited, but did not complete the study, of which five dogs were fed the MCTD and five were fed the placebo-standardised diet at the time of withdrawal. Of the five dogs that withdrew when on the MCTD, two were euthanised before completion of study due to uncontrollable cluster seizures. The remaining three withdrew due to the following reasons: dog becoming lethargic due to the diet; difficulty in feeding exclusively the test diet with a new puppy in the household; and dog not being satisfied with the diet, being constantly hungry and looking for food. Reasons for withdrawal of the five dogs on the placebo diet include the following: the study being too much a hassle; seizure activity increased with frequent cluster seizures; increased seizure frequency; owner unable to commit to the study due to other commitments; and food intolerance causing allergic reaction. One dog completed the study but was excluded from the analysis due to an error in the diet dispensed where the dog was given the same diet for both diet periods.

## Discussion

The primary focus of this study was to evaluate the antiepileptic efficacy and tolerability of a low MCT-containing diet to dogs with idiopathic epilepsy chronically treated with standard AEDs. To achieve this objective, twenty-one dogs with epilepsy were enroled into a 6-month prospective study, in which they were separately fed the MCTD containing low concentrations of MCT (5·5 % as fed) for 3 months and an isoenergetic placebo diet containing the same levels of fat, protein and carbohydrate for 3 months. The results show that, for the first time, the MCTD – a diet with low MCT inclusion rate – had positive effects on reduction of both seizure frequency and seizure day frequency per month. This KD was well tolerated and had no significant effect on weight or serum concentrations of glucose, PB or KBr in the dogs, and resulted in significantly higher serum concentrations of BHB, which was in accordance with the results reported by Patterson *et al.* ^(^ ^36^ ^)^ and Pan *et al.* ^(^ ^33^ ^)^. Collectively, the results support the use of the MCTD for difficult-to-manage canine epilepsy.

The KD diet is a therapeutic option commonly utilised in people with drug-resistant epilepsy, especially in children^(^ ^25^ ^)^. The anticonvulsant effectiveness of a KD, where most pharmacological treatments have failed, suggests a fundamental mechanistic difference between these treatment modalities. Although the exact mechanisms resulting in the antiepileptic effects of the KD remain elusive, proposed mechanisms include involvement and/or alterations in brain energy metabolism, neurotransmitters, ketone bodies, AED, fatty acids and neuroactive peptides^(^ ^37^ ^–^ ^40^ ^)^. In human clinical practice, during a KD treatment, it has been shown that serum AED concentrations may increase despite no changes in the dosage administered. In such cases, serum concentrations of PB have been shown to increase by up to 100 %^(^ ^41^ ^)^. It is, thus, reasonable to hypothesise an association between the antiepileptic effects of KD and the ability of the diet to influence serum concentrations of AEDs. This was investigated in an open clinical study of fifty-one children on a KD, which showed no significant changes in PB serum concentrations, along with other commonly administered AED^(^ ^42^ ^)^. The results presented in this study also show no significant changes in both the PB and the KBr plasma concentrations during the MCTD phase, suggesting alternative mechanisms of anticonvulsant properties.

The MCTD utilised in this study has previously been shown to improve the brain function of aged dogs, and was hypothesised to exert these cognitive enhancements by providing the brain with an alternative energy source^(^ ^33^ ^)^. Ageing is commonly associated with declined efficiency of glucose metabolism in the brain, and has been demonstrated in both animals and humans. The MCTD is able to increase ketone concentrations in the blood, and thus provides an alternative metabolic pathway^(^ ^33^ ^)^. Secondary to providing an alternative energy source, MCT may contribute to the maintenance of the neuronal structure via increasing concentrations of PUFA in the brain, which have also been shown to decrease as a consequence of ageing^(^ ^33^ ^)^. The MCTD described in this study shows positive effects in both cognitive enhancement and seizure control, where pathological mechanisms or pathways involved may be similar or may act in a synergistic manner.

KD have often been associated with increases in BHB and other ketone bodies such as acetone and acetoacetate, which is in accordance with this study where administration of the MCTD resulted in significant increases in serum concentrations of BHB^(^ ^33^ ^,^ ^43^ ^,^ ^44^ ^)^. It has, thus, been hypothesised that the ketone bodies play a crucial role in the antiepileptic properties seen in KD. Studies involving administration of acetone and acetoacetate in animal models including rabbits, mice and rats have shown protection against induced seizures to varying degrees^(^ ^45^ ^–^ ^48^ ^)^. In our study, we showed MCTD-related antiepileptic effects that were poorly correlated with relative changes in serum BHB concentrations. As serum concentrations of ketone bodies have not been consistently correlated with antiepileptic efficacy, others have suggested the importance of relative glucose reductions along with elevation of ketone bodies^(^ ^49^ ^)^. However, we showed reductions in seizure frequency with no significant changes in serum glucose concentrations between the MCTD and placebo diets. The MCTD in this study resulted in seizure control, manifesting as seizure frequency and seizure day frequency reductions, which were not correlated with age or weight. The mechanisms resulting in antiepileptic effects upon consumption of the MCTD seem to be irrespective of the age-related reduced efficiency of glucose metabolism or increases in relative serum BHB concentrations.

The results of this study suggest a rapid onset of antiepileptic properties associated with consumption of the MCTD. It is, therefore, likely that the mechanisms of action leading to the antiepileptic effects possessed by the MCTD also involve rapid biological processes. Studies have highlighted the potential involvement of individual constituents of the MCTKD in the antiepileptic properties associated with the effectiveness of a KD. The main constituents of the MCTKD consist of the 8-carbon caprylic acid along with other MCT including the 10-carbon capric acid and 12-carbon lauric acid^(^ ^50^ ^–^ ^52^ ^)^. Caprylic acid has been shown to significantly and dose-dependently increase the dosage of pentylenetetrazol required to induce myoclonic twitch and clonic convulsions^(^ ^50^ ^)^. Capric acid has also been shown to significantly and dose-dependently increase the seizure thresholds in the 6Hz and maximal electroshock seizure threshold tests^(^ ^51^ ^)^. It was, thus, suggested that other TAG may possess similar antiepileptic properties as those seen in caprylic and capric acids. Interestingly valporic acid (VPA, 2-propulpentanoic acid), the most common and widely used broad-spectrum epilepsy drug in humans worldwide, is also a TAG^(^ ^52^ ^)^. Unfortunately, VPA is commonly associated with unfavourable side-effects such as teratogenicity and hepatotoxicity. This highlights the need to search for novel agents such as fatty acid compounds with improved potency against seizures and a better side-effects profile compared with the drugs currently available. Recently, it has been shown that seizure activity correlates with phosphoinositide depletion and that VPA acts by restoring phosphoinositide levels^(^ ^53^ ^,^ ^54^ ^)^. Other MCT such as 4-methyloctanoic acid (hircinoic acid) and nonanoic acid (pelargonic acid) were shown to exhibit similar effects with evidence of enhanced seizure control and an improved side-effects profile in comparison with VPA^(^ ^52^ ^–^ ^55^ ^)^. It has been shown that a MCTKD causes not only increases in ketone body production but also accumulation of MCT in the blood plasma; therefore, it is possible that specific MCT or downstream metabolites are acting directly to exert antiepileptic effects^(^ ^52^ ^)^. Furthermore, the MCTD implemented in this study contains much higher proportions of carbohydrates in comparison with traditional KD used in humans, which contain ratios of about 4:1 or 3:1 fats to proteins and carbohydrates. Despite this difference, the MCTD diet still shows antiepileptic effects, highlighting a disassociation between antiepileptic effectiveness and specific diet ratios, but instead potentially suggesting involvement of specific metabolite agents or constituents, such as fatty acid compounds with antiepileptic properties. However, canine and human metabolism of fats and carbohydrates differ, as dogs rely more significantly on fat metabolism compared with humans. More research is necessary to elucidate the mechanistic differences in fat metabolism and the relationship of energitic proportions contributed by MCT in KD.

The placebo effect is a well-known phenomenon and has been demonstrated, manifesting as a decrease in seizure frequency, in dogs with epilepsy. It was shown that the placebo effect accounted for 26–46 % of seizure reductions during placebo administration in comparison with baseline values^(^ ^56^ ^)^. The placebo response is thought to originate from bias in the owners’ observations and data collection, which is influenced by the positive attitudes of owners towards respective intervention treatment. This study comprised of a triple-blinded, randomised cross-over diet trial design, where the placebo effect was not relevant as dogs were fed both the test diet and the placebo diet. Another factor besides the placebo effect is the regression to the mean effect, highlighting the fact that most diseases wax and wane and that recruitment peaks at the time when the clinical signs are more severe^(^ ^56^ ^)^. We, therefore, have disregarded the analysis of the baseline data, which was only used as an inclusion criterion for the prospective trial.

In conclusion, the data shown are in accordance with evidence in the literature showing antiepileptic properties of the MCTKD and KD, manifesting as reductions in seizure activities. By maintaining clinically effective levels of ketosis and anticonvulsant efficacy, this research indicates that more carbohydrates, although still a KD, can be utilised with the MCTKD to increase palatability, nutrient balance, compliance and acceptability of the diet. This study provides evidence for the therapeutic management of canine epilepsy using the MCTD with further potential implications in dietary management for both human and canine drug-resistant epilepsy. Further investigations into the effectiveness and efficacy of the MCTD on epilepsy should involve a pragmatic approach with larger randomised controlled trials and inclusion of an intention-to-treat analysis.

## Supplementary material

For supplementary material accompanying this paper visit http://dx.doi.org/10.1017/S000711451500313X. click here to view supplementary material
