# Supplementary material for: A randomised trial of a medium-chain TAG diet as treatment for dogs with idiopathic epilepsy
Source: Br J Nutr. 2015 Sep 4;114(9):1438–47. doi: 10.1017/S000711451500313X (PMC4635653; doi:10.1017/S000711451500313X)
Supplement: Supplementary file 1 [file S000711451500313Xsup001.doc]

**Supplementary Table 1**. Adverse events recorded for dogs during the standardised placebo diet () and MCTD ().Details of the adverse event includes: a description of the adverse event; length of adverse event recorded in days; severity of adverse event recorded using 1=mild, 2=moderate, 3=severe and 4=serious; frequency of adverse event measured using 1=once, 2=occasionally, 3=regularly and 4=on-going; concomitant treatment noted as 1=none and 2=yes and final outcomes consisted of 1=resolved without further effects, 2=resolved with further effects, 3=unchanged, 4=euthanasia and 5=death. Adverse events were recorded over a period of 3 months (±2 days) for the standardised placebo diet and 3 months (±2 days) for the MCTD

| **Cases** | **Adverse effects** | **Length of effect (days)** | **Severity** | **Frequency** | **Treatment** | **Outcome** |
| --- | --- | --- | --- | --- | --- | --- |
| **SCN02** | Vomiting (mild) | 1 | 1 | 1 | 1 | 1 |
|  | Vomiting (mild) | 1 | 1 | 1 | 1 | 1 |
|  | Vomiting (mild) | 1 | 1 | 1 | 1 | 1 |
| **SCN05** | Ear mite infestation | 13 | 3 | 1 | 2 | 1 |
|  | Dry skin | Ongoing | 1 | 4 | 2 | 3 |
| **SCN12** | Unusual behavioural abnormalities | 4 | 1 | 3 | 1 | 1 |
|  | Itchiness (ears) | 11 | 1 | 1 | 2 | 1 |
|  | Itchiness (general) | 3 | 1 | 2 | 1 | 1 |
|  | Vomiting (mild) | 1 | 1 | 1 | 1 | 1 |
|  | Cough (dry) | 4 | 1 | 1 | 1 | 1 |
|  | Itchiness (ears) | 8 | 1 | 1 | 2 | 1 |
| **SCN13** | Vomiting | 1 | 1 | 1 | 1 | 1 |
|  | Weeping in eyes | Ongoing | 1 | 4 | 1 | N/A |
| **SCN14** | Physical injury (toenail) | 6 | 1 | 1 | 2 | 1 |
|  | Vomiting | 3 | 1 | 1 | 1 | 1 |
| **SCN15** | None | None | None | None | None | None |
| **SCN16** | Diarrhoea | 5 | 2 | 3 | 1 | 1 |
| **SCN17** | Enlarged submandibular lymph nodes | 4 | 3 | 1 | 2 | 1 |
| **SCN19** | Urinary tract infection | 16 | 1 | 4 | 2 | 1 |
|  | Inappetant vomiting | 17 | 2 | 2 | 2 | 1 |
| **SCN20** | Diarrhoea | 4 | 1 | 1 | 2 | 1 |
| **SCN23** | Physical injury (fractured claw) and infection (claw) | 8 | 1 | 1 | 2 | 1 |
| **SCN25** | Burst anal gland abscess | 8 | 1 | 1 | 2 | 1 |
|  | Uncoordinated vague, pruritic, hyperactive | Ongoing | 1 | 4 | 1 | 3 |
|  | Infection (ear) | Ongoing | 2 | 1 | 2 | 3 |
|  | Diarrhoea | unknown | 1 | 2 | 2 | 1 |
| **SCN29** | Vomiting | 1 | 1 | 1 | 1 | 1 |
|  | Vomiting | 1 | 1 | 1 | 1 | 1 |
|  | Eosinophilia | 1 | 1 | 1 | 1 | 1 |
| **SCN30** | None | None | None | None | None | None |
| **SCN33** | Behavioural abnormalities | 2 | 1 | 1 | 2 | 1 |
|  | Diarrhoea | 2 | 1 | 1 | 2 | 1 |
|  | Conjunctivitis (Chalazion) | 20 | 1 | 1 | 2 | 1 |
|  | Surgery (Chalazion removal) | 11 | 1 | 1 | 2 | 1 |
|  | Surgery (ear wart removal) | 11 |  |  |  |  |
|  | Bilateral otitis | Ongoing | 1 | 1 | 2 |  |
|  | Conjunctivitis (both eyes) | 7 | 1 | 1 | 2 | 1 |
|  | **Adverse effects** | **Length of effect (days)** | **Severity** | **Frequency** | **Treatment** | **Outcome** |
| **SCN02** | Abnormal blood tests | 25 | 2 | 1 | 1 | 1 |
|  | Vomiting and pyrexia | 16 | 2 | 3 | 2 | 1 |
| **SCN09** | Small cyst-like growth on base of left ear pinnar | Ongoing | 1 | 1 | 1 | 3 |
|  | Plaque build up on teeth | Ongoing | 1 | 3 | 1 | 3 |
| **SCN12** | Ingestion of 2 metal bottle caps | 7 | 4 | 1 | 2 | 1 |
|  | Post-surgery complications (aspiration pneumonia) | 28 | 2 | 1 | 2 | 1 |
|  | Post-surgery complications (clipper rash) | 4 | 1 | 1 | 2 | 1 |
|  | Medication overdose (antibiotic) | 1 | 1 | 1 | 1 | 1 |
|  | Sore skin and ear infection | 70 | 1 | 3 | 2 | 1 |
|  | Diarrhoea | 5 | 1 | 1 | 2 | 1 |
| **SCN13** | Tenesmus and constipation | 7 | 2 | 3 | 2 | 2 |
| **SCN14** | Arthritis (pain worsened) | Ongoing | 1 | 3 | 2 | 3 |
| **SCN15** | Knuckling (forelimbs) | Ongoing | 1 | 2 | 1 | 3 |
| **SCN17** | Vomiting | 1 | 2 | 4 | 2 | 1 |
| **SCN20** | Conjunctivitis (mild) | 7 | 1 | 1 | 2 | 1 |
| **SCN21** | Diarrhoea | 10 | 2 | 2 | 2 | 1 |
| **SCN22** | Seizures (5 in one day) | 1 | 2 | 1 | 2 | 1 |
|  | Vomiting (bile present) | 1 | 1 | 1 | 1 | 1 |
|  | Lethargic and ataxia | 1 | 1 | 1 | 2 | 1 |
| **SCN23** | Heat stroke (vomiting, diarrhoea, haemophargic) | 7 | 3 | 1 | 2 | 1 |
| **SCN25** | Vomiting after Mexican food ingestion | 1 | 1 | 1 | 1 | 1 |
|  | Inflammation of anal gland | 13 | 1 | 1 | 1 | 3 |
| **SCN29** | Vomiting | 1 | 1 | 1 | 1 | 1 |
|  | Vomiting after chocolate ingestion | 1 | 1 | 1 | 1 | 1 |
|  | Oral warts | Ongoing | 1 | 1 | 1 | 3 |
|  | Eosinophilia | Ongoing | 1 | 1 | 1 | 1 |
| **SCN30** | Physical injury (cut to pad) | 11 | 1 | 1 | 1 | 1 |
| **SCN33** | N/A | N/A | N/A | N/A | N/A | N/A |

**Supplementary Table 2.** Concomitant treatment recorded for dogs during the standardised placebo diet () and MCTD (), detailing indications for treatment, products used and length of treatments. Concomitant treatments were recorded over a period of 3 months (±2 days) for the standardised placebo diet and 3 months (±2 days) for the MCTD

| **Cases** | **Indication for treatment** | **Products** | **Length of treatment (days)** |
| --- | --- | --- | --- |
| **SCN02** | Unknown vomiting and pyrexia | Synulox RTU | 1 |
|  | Pyrexia | Carprieve | 1 |
|  | Dehydration following vomiting | Lectade | 15 (as needed) |
|  | Pyrexia and vomiting | Nisamox | 7 |
| **SCN05** | Ear mites | Advocate (for extra large dogs) | 1 |
|  | Ear mites | Advocate (for extra large dogs) | 1 |
|  | Wormer (3 monthly) | Milbemax | 1 |
| **SCN06** | N/A | N/A | N/A |
| **SCN12** | Itchy skin | fuciderm carboner gel 15g | 5 |
|  | Sore ears | otomax eardrops | 7 |
|  | Stenosis (ears) | otomax eardrops Gentimicin | 7 |
|  | Itchy and sore skin | Dermisol 30g propyleneglycol 1.75% | 6 |
|  | Sore Ears | otomax ear drops , gentamicin | 8 |
|  | Diarrhoea | Prokolin | 5 |
|  | Itchy skin | Prednisolone 5mg | 4 |
|  | Itchy skin | Prednisolone 5mg | 5 |
|  | Analgesia | Tramadol 50mg | 6 |
|  | Antacid | Omeprazole 20mg capsules | 7 |
|  | Antibiotic | Olavaceptin 500mg amoxicillin - claulante | 14 |
|  | Clipper rash | Puciderm | 5 |
|  | Panacur for poss giardia | Penbenazole 4.5g | 4 |
|  | Removal (surgical) of bottle top parasite | methadone, midazolam, propotol | 1 |
|  | Antiemetic | maropitant, metaclopomide | 7 |
|  | Antacid | omeprazole 20mg capsules | 7 |
|  | Analgesia | buprenorphine | 7 |
|  | Appetite | mirtazapine | 7 |
|  | Itchy skin | prednisolone 5mg | Ongoing |
| **SCN13** | Back pain | PLT 200mg ci9nchophen, 1mg PREd | 30 |
|  | Worming | 75mg praziquiantel, 144mg pyrantel embonate, 150mg febantel | 1 |
|  | Gastrointestinal | metronidazone | Ongoing |
| **SCN14** | Arthritis relief | Previcox 227mg | Ongoing |
| **SCN15** | Vaccination | nobivac lepto 2 | 1 |
|  | Vaccination | nobivac DHP | 1 |
| **SCN16** | Worming | Panacur | 1 |
|  | Worming | Drontal plus | 1 |
|  | Flea treatment | frontline combo | 1 |
|  | Worming | advocate | 1 |
| **SCN17** | Vomiting | Carorieve (carprofen) | 1 |
|  | Worming | milbemax | 1 |
| **SCN19** | Flea treatment | Frontline Fipronil 134mg / methaprene 120.6mg | 1 |
|  | Vaccination | VBVanguard 7 | 1 |
| **SCN20** | Conjunctivitis | Fucithalmic | 8 |
| **SCN21** | Flea treatment | advocate >10kg | 1 |
|  | Flea treatment | advocate >10kg | 1 |
|  | Diarrhoea (antibiotics given) | Amoxycillin LA | 1 |
|  | Diarrhoea (antibiotics given) | Noroclaw | 14 |
|  | Diarrhoea | Pro-kolin paste | 7 |
| **SCN22** | Suspected adverse reaction to Imepitoin | Fluids | 1 |
| **SCN23** | N/A | N/A | N/A |
| **SCN25** | Worming | Drontal | 1 |
| **SCN27** | Flea treatment | Advocate Imidaclopid | 1 |
|  | Worming | Drontal | 1 |
| **SCN28** | Liver health | Milk thistle | Ongoing |
| **SCN29** | Flea and worm treatment | Advocate | 1 |
|  | Flea and worm treatment | Advocate | 1 |
|  | Flea treatment | Stronghold | 1 |
|  | Worming | Drontal Plus | 1 |
| **SCN30** | Physical injury (cut to pad) | Metacam | 7 |
|  | Physical injury (cut to pad) | Metacam | 1 |
| **Cases** | **Indication for treatment** | **Products** | **Length of treatment (days)** |
| **SCN02** | Unknown vomiting and pyrexia | Synulox RTU | 1 |
|  | Pyrexia | Carprieve | 1 |
|  | Dehydration following vomiting | Lectade | 15 (as needed) |
|  | Pyrexia and vomiting | Nisamox | 7 |
| **SCN05** | Ear mites | Advocate (for extra large dogs) | 1 |
|  | Ear mites | Advocate (for extra large dogs) | 1 |
|  | Wormer (3 monthly) | Milbemax | 1 |
| **SCN06** | N/A | N/A | N/A |
| **SCN12** | Itchy skin | fuciderm carboner gel 15g | 5 |
|  | Sore ears | otomax eardrops | 7 |
|  | Stenosis (ears) | otomax eardrops Gentimicin | 7 |
|  | Itchy and sore skin | Dermisol 30g propyleneglycol 1.75% | 6 |
|  | Sore Ears | otomax ear drops , gentamicin | 8 |
|  | Diarrhoea | Prokolin | 5 |
|  | Itchy skin | Prednisolone 5mg | 4 |
|  | Itchy skin | Prednisolone 5mg | 5 |
|  | Analgesia | Tramadol 50mg | 6 |
|  | Antacid | Omeprazole 20mg capsules | 7 |
|  | Antibiotic | Olavaceptin 500mg amoxicillin - claulante | 14 |
|  | Clipper rash | Puciderm | 5 |
|  | Panacur for poss giardia | Penbenazole 4.5g | 4 |
|  | Removal (surgical) of bottle top parasite | methadone, midazolam, propotol | 1 |
|  | Antiemetic | maropitant, metaclopomide | 7 |
|  | Antacid | omeprazole 20mg capsules | 7 |
|  | Analgesia | byprenorphine | 7 |
|  | Appetite | mirtazapine | 7 |
|  | Itchy skin | prednisolone 5mg | Ongoing |
| **SCN13** | Back pain | PLT 200mg ci9nchophen, 1mg PREd | 30 |
|  | Worming | 75mg praziquiantel, 144mg pyrantel embonate, 150mg febantel | 1 |
|  | Gastrointestinal | metronidazone | Ongoing |
| **SCN14** | Arthritis relief | Previcox 227mg | Ongoing |
| **SCN15** | Vaccination | nobivac lepto 2 | 1 |
|  | Vaccination | nobivac DHP | 1 |
| **SCN16** | Worming | Panacur | 1 |
|  | Worming | Drontal plus | 1 |
|  | Flea treatment | frontline combo | 1 |
|  | Worming | advocate | 1 |
| **SCN17** | Vomiting | Carorieve (carprofen) | 1 |
|  | Worming | milbemax | 1 |
| **SCN19** | Flea treatment | Frontline Fipronil 134mg / methaprene 120.6mg | 1 |
|  | Vaccination | VBVanguard 7 | 1 |
| **SCN20** | Conjunctivitis | Fucithalmic | 8 |
| **SCN21** | Flea treatment | advocate >10kg | 1 |
|  | Flea treatment | advocate >10kg | 1 |
|  | Diarrhoea (antibiotics given) | Amoxycillin LA | 1 |
|  | Diarrhoea (antibiotics given) | Noroclaw | 14 |
|  | Diarrhoea | Pro-kolin paste | 7 |
| **SCN22** | Suspected adverse reaction to Imepitoin | Fluids | 1 |
| **SCN23** | N/A | N/A | N/A |
| **SCN25** | Worming | Drontal | 1 |
| **SCN27** | Flea treatment | Advocate Imidaclopid | 1 |
|  | Worming | Drontal | 1 |
| **SCN28** | Liver health | Milk thistle | Ongoing |
| **SCN29** | Flea and worm treatment | Advocate | 1 |
|  | Flea and worm treatment | Advocate | 1 |
|  | Flea treatment | Stronghold | 1 |
|  | Worming | Drontal Plus | 1 |
| **SCN30** | Physical injury (cut to pad) | Metacam | 7 |
|  | Physical injury (cut to pad) | Metacam | 1 |
| **SCN33** | N/A | N/A | N/A |

**Supplementary Table 3**. Age, sex, weight, breed and neuter status of the all dogs included in this study.

| **Dog SCN** | **Dog breed** | **Age (years.months)** | **Weight (kg)** | **Sex (M/F)** | **Neuter status (Y/N)** |
| --- | --- | --- | --- | --- | --- |
| **02** | English Springer Spaniel | 5.5 | 21.60 | FEMALE | YES |
| **15** | Boxer | 6.2 | 30.15 | FEMALE | NO |
| **17** | English Bull Terrier | 2.4 | 29.90 | FEMALE | YES |
| **20** | Cross breed | 2.2 | 25.90 | FEMALE | YES |
| **23** | Saint Bernard | 3.8 | 62.40 | FEMALE | NO |
| **28** | Ihasn Aspo | 3.0 | 9.45 | FEMALE | YES |
| **05** | Golden retriever | 3.2 | 38.90 | MALE | NO |
| **06** | Cross breed | 2.2 | 26.50 | MALE | YES |
| **09** | Rhodesian Ridgeback | 4.3 | 46.30 | MALE | NO |
| **12** | Welsh Springer Spaniel | 4.3 | 25.15 | MALE | YES |
| **13** | Beagle | 6.8 | 14.95 | MALE | YES |
| **14** | American Bulldog | 4.3 | 33.80 | MALE | YES |
| **16** | Border Collie | 4.6 | 26.70 | MALE | NO |
| **19** | Border Collie | 4.3 | 17.30 | MALE | YES |
| **21** | Cavalier King Charles | 4.7 | 10.20 | MALE | YES |
| **22** | Mastiff | 5.7 | 63.70 | MALE | YES |
| **25** | German Shepherd | 3.8 | 36.75 | MALE | NO |
| **27** | Cross breed | 9.7 | 13.50 | MALE | YES |
| **29** | Siberian Huskey | 4.2 | 34.10 | MALE | YES |
| **30** | Beagle | 5.9 | 21.30 | MALE | NO |
| **33** | Slovakian Rough Haired Pointer | 5.5 | 36.95 | MALE | YES |

**Supplementary Table 4**. Overview of seizure frequency per month and seizure day frequency per month in each dog during the placebo diet and MCTD respectively (n=21). Overall response is represented by the percentage change between outcome variables respectively. Wilcoxon matched paired t-tests were used to make comparisons between diet groups. Statistical significances between diets are presented as p-values and highlighted in blue

|  | **Seizure frequency per month (p=0.020)** | | |  | **Seizure day frequency per month (p=0.022)** | | |
| --- | --- | --- | --- | --- | --- | --- | --- |
| **DOG SCN** | **Placebo** | **MCTD** | **Change (%)** | **DOG SCN** | **Placebo** | **MCTD** | **Change (%)** |
| **02** | 2.02 | 0.00 | -100.0 | **15** | 1.69 | 0.00 | -100.0 |
| **12** | 0.65 | 0.00 | -100.0 | **29** | 0.65 | 0.00 | -100.0 |
| **15** | 0.33 | 0.00 | -100.0 | **33** | 0.33 | 0.00 | -100.0 |
| **28** | 2.28 | 0.66 | -71.1 | **23** | 4.04 | 1.00 | -75.3 |
| **25** | 2.64 | 1.00 | -62.1 | **28** | 1.30 | 0.33 | -74.7 |
| **23** | 6.07 | 2.33 | -61.5 | **02** | 1.67 | 0.67 | -59.6 |
| **17** | 22.92 | 9.89 | -56.9 | **09** | 1.58 | 0.67 | -57.8 |
| **06** | 11.61 | 5.67 | -51.2 | **25** | 1.98 | 1.00 | -49.4 |
| **21** | 10.57 | 5.20 | -50.8 | **17** | 13.82 | 7.58 | -45.1 |
| **20** | 2.67 | 1.33 | -50.0 | **27** | 4.04 | 2.31 | -42.9 |
| **09** | 1.89 | 1.00 | -47.2 | **19** | 3.09 | 1.98 | -36.0 |
| **13** | 1.67 | 1.01 | -39.3 | **21** | 7.50 | 4.90 | -34.7 |
| **27** | 5.39 | 3.30 | -38.9 | **14** | 3.51 | 2.33 | -33.5 |
| **19** | 3.71 | 2.31 | -37.8 | **30** | 2.70 | 2.00 | -25.8 |
| **14** | 3.51 | 2.33 | -33.5 | **20** | 1.67 | 1.33 | -20.0 |
| **22** | 3.63 | 3.91 | 7.9 | **22** | 1.98 | 1.63 | -17.6 |
| **16** | 2.02 | 2.28 | 12.9 | **16** | 1.01 | 0.98 | -3.3 |
| **30** | 3.37 | 5.00 | 48.3 | **06** | 1.61 | 2.00 | 24.0 |
| **29** | 1.35 | 2.33 | 73.1 | **05** | 2.39 | 4.00 | 67.6 |
| **05** | 4.43 | 8.00 | 80.5 | **13** | 1.01 | 2.33 | 130.7 |
| **33** | 0.34 | 1.67 | 388.9 | **12** | 0.34 | 1.67 | 388.9 |

**Supplemental table 5**. Correlation coefficient analysis between seizure frequency reduction and seizure day frequency reduction with age, weight and BHB concentrations (n=21)

| **Variables compared in Pearsons correlation coefficient analysis** | **Statistical significance (p value)** | **Pearson’s correlation (r value)** |
| --- | --- | --- |
| Age vs Seizure frequency per month reduction | 0.658 | 0.0105 |
| Age vs Seizure day frequency per month reduction | 0.908 | -0.0270 |
| BHB (MCTD) vs Seizure frequency per month (MCTD) | 0.748 | -0.0744 |
| BHB (MCTD) vs Seizure day frequency per month (MCTD) | 0.583 | -0.127 |
| BHB (Placebo) vs Seizure frequency per month (Placebo) | 0.948 | 0.0152 |
| BHB (Placebo) vs Seizure day frequency per month (Placebo) | 0.868 | -0.0387 |
| BHB (difference) vs Seizure frequency per month reduction | 0.321 | 0.228 |
| BHB (difference) vs Seizure day frequency per month reduction | 0.147 | -0.327 |
| Weight (average) vs Seizure frequency per month reduction | 0.357 | 0.212 |
| Weight (average) vs Seizure day frequency per month reduction | 0.506 | -0.154 |
| Weight (MCTD) vs Seizure frequency per month (MCTD) | 0.935 | -0.0190 |
| Weight (MCTD) vs Seizure day frequency per month (MCTD) | 0.404 | -0.192 |
| Weight (Placebo) vs Seizure frequency per month (Placebo) | 0.766 | -0.0690 |
| Weight (Placebo) vs Seizure day frequency per month (Placebo) | 0.693 | -0.0916 |
